# Leveraging telemedicine for enhanced maternal and perinatal care in rural Nepal: opportunities and challenges

**DOI:** 10.1097/MS9.0000000000002588

**Published:** 2024-09-23

**Authors:** Pratik Adhikari

**Affiliations:** B.P. Koirala Institute of Health Sciences, Dharan, Nepal

**Keywords:** maternal, perinatal health, rural healthcare, telemedicine

## Abstract

This article explores the potential of telemedicine to revolutionize maternal and perinatal healthcare delivery in rural Nepal, where access to medical facilities is limited. By utilizing telemedicine technologies like remote consultations, mobile health applications, and tele-ultrasound, the authors can overcome barriers to healthcare access and improve outcomes for expectant mothers and newborns. The paper assesses the opportunities presented by telemedicine interventions and addresses the challenges that must be addressed for successful integration into rural healthcare systems.

## Introduction

Highlights

- Telemedicine can significantly improve maternal and perinatal health outcomes in rural Nepal by overcoming geographical and infrastructural barriers.
- Successful telemedicine initiatives globally provide a valuable framework for implementing similar interventions in rural Nepal.
- Addressing challenges such as internet connectivity, training, and regulatory frameworks is crucial for effective telemedicine integration.
- Collaborative efforts among government agencies, healthcare organizations, and technology developers are essential for the sustainable adoption of telemedicine.

In rural Nepal, maternal and perinatal health outcomes are significantly challenged by a shortage of medical facilities, geographical barriers, and a lack of skilled healthcare providers. According to the Nepal Demographic and Health Survey (NDHS) 2022, only 60% of births in rural areas are attended by skilled health personnel, compared to 89% in urban areas^1^. This disparity contributes to Nepal’s maternal mortality rate (MMR) of 151 per 100 000 live births, which, while improved, still highlights significant challenges compared to the global average^2^. Telemedicine offers a promising solution by facilitating remote access to healthcare services and expertise, thereby transforming the delivery of maternal and perinatal care.

In countries such as India and Bangladesh, where similar geographical and resource challenges exist, telemedicine has shown positive impacts. A study in rural India reported a 20% reduction in maternal mortality due to telemedicine interventions^3^. Similarly, Bangladesh’s telemedicine program has led to a 30% increase in antenatal care visits^4^. By connecting expectant mothers and healthcare providers through digital platforms, telemedicine has the potential to bridge the gap in healthcare access and improve outcomes for rural communities. By learning from these regions, Nepal can adapt telemedicine strategies to its unique challenges and improve maternal and perinatal healthcare outcomes.

### Literature review

Recent studies underscore the effectiveness of telemedicine in enhancing maternal and perinatal health outcomes. Mehta *et al*. (2023) conducted a randomized controlled trial (RCT) involving 500 pregnant women in low-resource settings to evaluate the impact of mobile health technology. The study employed a mixed-methods approach, including quantitative surveys and qualitative interviews, to assess changes in maternal and child healthcare access and utilization. The findings highlighted significant improvements in antenatal care visits and maternal knowledge regarding pregnancy-related issues.

Similarly, Memon *et al*. (2022) conducted a systematic review and meta-analysis of 25 studies across various low-income countries and middle-income countries. The review followed PRISMA guidelines to ensure a comprehensive and unbiased selection of studies. The meta-analysis focused on measuring the effect size of telemedicine interventions on maternal and neonatal mortality rates. The results demonstrated a statistically significant reduction in mortality rates, emphasizing the critical role telemedicine plays in healthcare delivery^5^.

These findings underscore the significance of leveraging telemedicine technologies to address healthcare disparities in rural Nepal. The rigorous methodologies employed in these studies provide robust evidence supporting the potential benefits of telemedicine interventions^6^.

### Methodological approach

This review article synthesizes information from various sources, including case studies, interviews with healthcare professionals, and observations from telemedicine program implementations. The case studies were selected based on specific criteria, such as the geographic area, type of telemedicine intervention, and the duration of program implementation. Semi-structured interviews with healthcare professionals were conducted using purposive sampling to gather insights from those directly involved in telemedicine initiatives. The information gathered provides a comprehensive understanding of the socio-technical aspects of telemedicine in rural Nepal, focusing on the Jhapa, Ilam, and Morang districts. Data triangulation was used to ensure the reliability and validity of the findings by comparing multiple data sources and perspectives. This approach offers a nuanced view of the challenges and opportunities associated with telemedicine in these regions.

### Informants and sites

The review incorporates insights from healthcare professionals and policymakers involved in telemedicine programs. The perspectives were gathered from sites as shown in Table 1 below.

**Table 1:** Distribution of informants by site and type in the study on healthcare management in eastern Nepal.

Site	Number of informants	Type of informants
Provincial hospital Bhadrapur	6	Healthcare professionals
District hospital Ilam	5	Healthcare professionals
District Health Office, Jhapa	4	Healthcare professionals
Ministry of Health and Population (MoHP)	1	Policymaker
Department of Health Service (DoHS)	1	Policymaker

These insights were collected through semi-structured interviews, site observations, and discussions, focusing on the implementation, and outcomes of telemedicine programs.

### Examples of telemedicine in Rural Nepal

#### Example 1: Jhapa

A pregnant woman from Jhapa experienced complications during her pregnancy. Due to the distance and financial constraints, she could not visit a specialist in a major city. However, through the telemedicine program, she visited the district hospital in Jhapa and participated in a videoconference consultation with a maternal-fetal medicine specialist from a hospital in Kathmandu. The intervention included a virtual ultrasound examination, real-time monitoring of vital signs, and collaborative decision-making between the local healthcare team and the specialist. The specialist provided a detailed care plan, including medication adjustments, lifestyle modifications, and scheduled follow-up appointments. Over the next few months, the woman’s condition improved, and she successfully gave birth to a healthy baby. The case resulted in a reduction of potential maternal complications and demonstrated improved adherence to the recommended care plan.

This case demonstrates how telemedicine enabled a pregnant woman from a remote area to access specialist maternal care at the district hospital, saving travel time and costs. A respondent from Jhapa mentioned that patient flow in the hospital has increased since the program’s implementation, as it has empowered the hospital team to provide necessary medical assistance. They stated: ‘Telemedicine programs, particularly videoconferencing, have been instrumental in offering medical support and ensuring safe deliveries’. They emphasized that the services should be user-friendly so that all health workers can use them easily. However, the respondents expressed dissatisfaction with frequent power cuts and internet interruptions, causing delays in sending emails and supporting documents for specialist consultations. Paramedics and nursing staff highlighted the importance of good computer skills, knowledge of medical terminologies, and reasonably good English for proper communication with specialists.

#### Example 2: Ilam

A young mother in Ilam was experiencing complications with her newborn baby. The local health post did not have the necessary equipment or specialists to provide appropriate care. One day, the mother took her baby to the district hospital in Ilam, where they used telemedicine services to connect with a neonatologist in Pokhara. The intervention involved a thorough review of the baby’s medical history, virtual physical examination through video, and remote monitoring of vital signs. The specialist recommended a comprehensive treatment plan, including medication, feeding practices, and guidelines for monitoring the baby’s progress. After several follow-up consultations, the baby’s health improved, and the mother felt more confident in caring for her newborn. The intervention led to improved neonatal health outcomes, with a noticeable decrease in hospital readmission rates.

This example shows how telemedicine can bridge the gap between remote areas and specialist services, ensuring timely and effective treatment for maternal and perinatal care. A respondent from Ilam stated that the telemedicine program has increased patient visits to the hospital, as it provides access to necessary medical assistance. They said: ‘Videoconferencing has proven to be the most effective method for obtaining medical support and treating mothers and newborns through the telemedicine program’. They noted the need for user-friendly services to ensure that all health workers can use the technology easily. However, respondents at rural hospitals mentioned challenges such as frequent power cuts and internet interruptions, which delay communication with specialists. Paramedics and nursing staff stressed the need for good computer skills, medical terminology knowledge, and reasonably good English for effective communication.

#### Example 3: Morang

An expectant mother in Morang was diagnosed with gestational diabetes. The nearest hospital lacked an endocrinologist, and her family could not afford to travel to a distant city for specialized care. Through the district hospital in Morang, she accessed telemedicine services and connected with an endocrinologist from a hospital in Biratnagar. The intervention included regular virtual consultations for diabetes management, personalized dietary counseling, and continuous glucose monitoring through telemedicine devices. The specialist assessed her condition via videoconference and prescribed medication, dietary changes, and regular monitoring of blood sugar levels. The specialist assessed her condition via videoconference and prescribed medication, dietary changes, and regular monitoring of blood sugar levels. Over the course of her pregnancy, the mother followed the treatment plan, and both she and her baby remained healthy. The intervention resulted in better glycemic control and a reduced risk of gestational diabetes-related complications.

This scenario illustrates how telemedicine can provide specialist maternal care to patients in remote areas, reducing the need for travel and associated costs. A respondent from Morang mentioned that the hospital has seen an increase in patient visits since the telemedicine program began, as it enables the hospital team to access necessary medical assistance. They stated: ‘Among the telemedicine program’s applications, videoconferencing has been the most effective in providing medical support and treating expectant mothers’. They highlighted the importance of user-friendly services so that all health workers can easily use the technology. However, respondents expressed concerns about frequent power cuts and internet disruptions, which cause delays in sending emails and supporting documents for specialist consultations. Paramedics and nursing staff emphasized the need for good computer skills, medical terminology knowledge, and reasonably good English to communicate effectively with specialists.

### Challenges and considerations

Despite the promise of telemedicine, several challenges must be addressed to ensure its successful implementation in rural Nepal. These challenges include limited internet connectivity, inadequate infrastructure, healthcare provider training, regulatory hurdles, and cultural acceptance. In regions like rural India and Bangladesh, similar challenges have been overcome by implementing solar-powered internet solutions, which ensure reliable connectivity in areas with frequent power cuts. Collaborative training programs for healthcare providers have also been established, focusing on technology use and cultural competency.

A deeper exploration of cultural challenges reveals the importance of addressing language barriers, varying levels of trust in digital health solutions, and differing perceptions of telemedicine’s effectiveness. In Nepal, efforts to incorporate local languages into telemedicine platforms and involve community leaders in promoting the benefits of telemedicine have been initiated to enhance cultural acceptance. Addressing these challenges requires a collaborative approach involving government agencies, healthcare organizations, technology developers, and community stakeholders.

### Recommendations

To harness the full potential of telemedicine for maternal and perinatal care in rural Nepal, stakeholders should prioritize the following recommendations:

1. Improve internet connectivity and infrastructure in rural areas to facilitate telemedicine consultations.
2. Provide comprehensive training programs for healthcare professionals, including midwives, on telemedicine technologies and protocols.
3. Develop robust regulatory frameworks to govern telemedicine usage and ensure patient privacy and data security.
4. Foster partnerships between government agencies, healthcare organizations, technology developers, and international donors to fund and support telemedicine initiatives.
5. Conduct regular evaluations and assessments to monitor the impact of telemedicine interventions and make necessary adjustments to optimize effectiveness and sustainability.

## Conclusion

Telemedicine holds immense promise for enhancing maternal and perinatal care in rural Nepal by overcoming barriers to access and improving healthcare outcomes for rural communities. By addressing challenges and implementing recommendations, stakeholders can leverage telemedicine to bridge the gap in healthcare access and improve maternal and perinatal health outcomes in rural Nepal.

## Ethical approval

Since the study is a review study, we did not obtain ethical approval.

## Consent

Not applicable.

## Source of funding

Not applicable.

## Author contribution

P.A.: provided the data and materials from the archive and his notes. wrote the manuscript, reviewed the manuscript, and did final editing.

## Conflicts of interest disclosure

The author declares no conflict of interest.

## Research registration unique identifying number (UIN)

This is a cross-sectional involving a human subject, so registration of research study was done.

1. Registry used: Researchregistry.com.
2. Unique identifying number or registration ID: researchregistry10355.

## Guarantor

Pratik Adhikari is the guarantor of the study.

## Data availability statement

The datasets supporting the conclusions of this article are included within the article.

## Provenance and peer review

Not commissioned or externally peer-reviewed.
